# Metabolic Feedback Inhibition Influences Metabolite Secretion by the Human Gut Symbiont Bacteroides thetaiotaomicron

**DOI:** 10.1128/mSystems.00252-20

**Published:** 2020-09-01

**Authors:** Jennie L. Catlett, Jonathan Catazaro, Mikaela Cashman, Sean Carr, Robert Powers, Myra B. Cohen, Nicole R. Buan

**Affiliations:** a Department of Biochemistry, University of Nebraska—Lincoln, Lincoln, Nebraska, USA; b Department of Chemistry, University of Nebraska—Lincoln, Lincoln, Nebraska, USA; c Department of Computer Science, Iowa State University, Ames, Iowa, USA; d Oak Ridge National Laboratory, Oak Ridge, Tennessee, USA; e Nebraska Center for Integrated Biomolecular Communication, Lincoln, Nebraska, USA; University of Delhi

**Keywords:** *Bacteroides*, acetate, *Bacteroides thetaiotaomicron*, formate, metabolism, secretion, microbiome, fermentation, anaerobic, bacteria, NMR metabolomics

## Abstract

Microbial metabolism and trophic interactions between microbes give rise to complex multispecies communities in microbe-host systems. Bacteroides thetaiotaomicron (*B. theta*) is a human gut symbiont thought to play an important role in maintaining host health. Untargeted nuclear magnetic resonance metabolomics revealed *B. theta* secretes specific organic acids and amino acids in defined minimal medium. Physiological concentrations of acetate and formate found in the human intestinal tract were shown to cause dose-dependent changes in secretion of metabolites known to play roles in host nutrition and pathogenesis. While secretion fluxes varied, biomass yield was unchanged, suggesting feedback inhibition does not affect metabolic bioenergetics but instead redirects carbon and energy to CO_2_ and H_2_. Flux balance analysis modeling showed increased flux through CO_2_-producing reactions under glucose-limiting growth conditions. The metabolic dynamics observed for *B. theta*, a keystone symbiont organism, underscores the need for metabolic modeling to complement genomic predictions of microbial metabolism to infer mechanisms of microbe-microbe and microbe-host interactions.

**IMPORTANCE** *Bacteroides* is a highly abundant taxon in the human gut, and Bacteroides thetaiotaomicron (*B. theta*) is a ubiquitous human symbiont that colonizes the host early in development and persists throughout its life span. The phenotypic plasticity of keystone organisms such as *B. theta* is important to understand in order to predict phenotype(s) and metabolic interactions under changing nutrient conditions such as those that occur in complex gut communities. Our study shows *B. theta* prioritizes energy conservation and suppresses secretion of “overflow metabolites” such as organic acids and amino acids when concentrations of acetate are high. Secreted metabolites, especially amino acids, can be a source of nutrients or signals for the host or other microbes in the community. Our study suggests that when metabolically stressed by acetate, *B. theta* stops sharing with its ecological partners.

## Abstract (precis)

*Bacteroides* is a highly abundant taxon in the human gut, and Bacteroides thetaiotaomicron (*B. theta*) is a ubiquitous human symbiont that colonizes the host early in development and persists throughout its life span. The phenotypic plasticity of keystone organisms such as *B. theta* is important to understand in order to predict phenotype(s) and metabolic interactions under changing nutrient conditions such as those that occur in complex gut communities. Our study shows *B. theta* prioritizes energy conservation and suppresses secretion of “overflow metabolites” such as organic acids and amino acids when concentrations of acetate are high. Secreted metabolites, especially amino acids, can be a source of nutrients or signals for the host or other microbes in the community. Our study suggests that when metabolically stressed by acetate, *B. theta* stops sharing with its ecological partners.

## INTRODUCTION

Microbes, whether in the environment or associated with host organisms, form complex multispecies communities that cooperate and compete to metabolize nutrients. The host gut ecosystem is a constantly changing landscape where symbiont organisms manage to establish long-term colonization despite the fact that the host regularly ingests and eliminates nutrients and transient microbes. This results in a constantly fluctuating environment where diverse microbes are secreting metabolic fermentation products and other secondary metabolic chemicals that may inhibit or stimulate neighboring organisms as they compete for nutrients. While there is recognition that microbes play important roles in host nutrition, health, and disease (1), it is difficult to conceptualize how diverse microbes interact with each other and the host in such a way as to be able to develop treatments or recommendations that preserve host-symbiont and beneficial microbe-microbe interactions while disfavoring pathogens.

Considering that bacterial virulence factors are often triggered by nutritional limitation (carbon, nitrogen, phosphorous, iron, etc.) (2–4) or physical stress (temperature, oxidative burst, etc.) (5, 6) and, at the molecular level, cause changes in intracellular metabolic flux and redox state/energy charge (7), the availability of nutrients and the physical factors that influence metabolism are at the crux of whether microbes induce virulence factors. Virulence factors such as cell invasion, chemotaxis, and siderophore and antibiotic synthesis, among others, can be recast as “nutrient searching” behaviors that are triggered by changes in the environment that result in decreased intracellular metabolic fluxes (8). Quorum sensing, in which bacteria secrete a small molecule that triggers expression of community-level behavior (sporulation, adherence, virulence, etc.) when it reaches a critical concentration (9), can also be “eavesdropped” by neighboring organisms in anticipation of intensification of competition for nutrients (10).

Symbionts have evolved to cooperate with hosts to establish long-term colonization strategies that do not result in disease and even protect the host from pathogens. It has been shown that establishing host-symbiont trophic relationships protects hosts from virulent interlopers by physically and nutritionally limiting the ability of pathogens to establish infections (11, 12), stimulating gut epithelial growth (13), and also modulating local immune response to maintain a healthy state (14, 15). It is hypothesized that perturbations of symbiont bacterial metabolism, such as through diet or antibiotic use, can disrupt this natural defensive relationship and predispose the host to disease by allowing pathogens to gain a metabolic foothold (16). By this reasoning, the dynamic interplay of nutrition and metabolism of colonizing symbiont bacteria (17) is crucial as they form the foundation of the host microbiome community with which transient and pathogenic microbes must compete for survival.

*Bacteroides* species are Gram-negative bacteria that are especially adept at metabolizing complex carbohydrates (18) and are often the dominant bacterial phylum in the digestive systems of many herbivorous and omnivorous animals, including humans (19). Bacteroides thetaiotaomicron (*B. theta*) is a nonpathogenic human gut symbiont that colonizes infants within a day of normal birth (20, 21). While *B. theta* is classified as nonpathogenic and has been shown to protect the host from *Salmonella* infection (22), it was shown to exacerbate infection by Citrobacter rodentium in a mouse model of enterohemorrhagic Escherichia coli (EHEC) disease (23), underscoring the complicated contributions of symbiont microbes to human health and disease. *B. theta* is closely related to sister species (24) that are implicated in irritable bowel disease (25) and periodontal disease (26) and has also been shown to carry and transmit antibiotic resistance genes through profligate conjugation with other bacteria (27, 28).

It was previously shown that *B. theta* secretes acetate, formate, propionate, and succinate into culture medium (29–31). *Bacteroides* species have been shown to produce low-molecular-weight heat-stable compounds that impair host defense by inhibiting migration and killing polymorphonuclear leukocyte (PMN) phagocytes (32, 33). Succinate and propionate, as low-molecular-weight heat-stable metabolites, have been hypothesized to fit the description and were shown to irreversibly inhibit superoxide and hydrogen peroxide production by neutrophils by lowering cytoplasmic pH (34). Propionate-secreting *B. theta* have also been shown to protect mice from colonization by *Salmonella*, presumably due to the same membrane-permeable pH-lowering property that is inherent to short-chain fatty acids (22). Acetate is also a membrane permeable (35) “switch” that reduces ATP synthesis in E. coli and regulates expression of virulence genes in many bacteria (36). Secretion of acetate, formate, propionate, and succinate by *B. theta* is therefore proposed to reduce the effectiveness of host response to pathogens and to have species-specific effects (enhancing or abrogating) bacterial colonization and virulence.

*B. theta* secretes metabolites as a result of starch, extracellular matrix, or glucose metabolism (18). *B. theta* catabolizes glucose via the Embden-Meyerhof-Parnas (EMP) pathway (glycolysis) to pyruvate, which is a major intracellular metabolite used as the substrate in gluconeogenesis, the tricarboxylic acid cycle (TCA cycle), and for biosynthesis of acetyl coenzyme A (acetyl-CoA), enzyme cofactors, and amino acids. Acetate, the major secreted product, can be synthesized with ATP using two metabolic pathways: (i) by hydrolysis of the CoA thioether bond by acetyl-CoA synthase (Acs) in the acetyl-CoA pathway, or (ii) by phosphotransacetylase (Pta) and acetate kinase (Ack) enzymes in the Ack/Pta pathway (see Table S1 in the supplemental material). The high concentration of secreted acetate suggests acetate is the primary energy-conserving overflow (37) by-product of *B. theta*. The second most abundant secreted product is succinate, which is produced by hydrolysis of succinyl-CoA by succinyl-CoA synthetase with generation of ATP in the TCA cycle. In the forward TCA cycle direction, succinate is funneled to succinate dehydrogenase, which oxidizes succinate to fumarate with the generation of reduced ubiquinone for generating a transmembrane proton gradient for ATP synthesis. These data suggest rapidly growing *B. theta* cells are limited in the turnover rate of reduced/oxidized quinone and secrete succinate as an intermediate product to maintain rates of glycolysis and glucose consumption (38). In the reverse TCA direction, succinate synthesis requires ATP and HCO_3_^−^ (pyruvate carboxylase), NADH (malate dehydrogenase), and reduced quinone (succinate dehydrogenase), and though enzyme steps are reversible, succinate synthesis by reverse TCA can only occur when there is a surplus of ATP generated as a result of forward TCA pathway flux.

10.1128/mSystems.00252-20.3 TABLE S1 Student’s *t* test *P* values for metabolomics data in Fig. 3d. *P* values are given for each metabolite measured in spent medium as acetate is supplemented from 0 to 10 mM. Download Table S1, DOCX file, 0.1 MB. Copyright © 2020 Catlett et al. 2020 Catlett et al. This content is distributed under the terms of the Creative Commons Attribution 4.0 International license.

The next most abundant secreted products are formate and propionate. Formate is synthesized by pyruvate formate-lyase, which uses pyruvate and coenzyme A as the substrates to produce formate and acetyl-CoA. Formate is therefore an energy neutral overflow metabolite that nevertheless increases the enzymatic routes to acetyl-CoA. Propionate is synthesized from succinyl-CoA to propionyl-CoA by methylmalonyl-CoA mutase, methylmalonyl-CoA epimerase, and propionyl-CoA carboxylase enzymes, with subsequent thioesterase activity by the same Acs or Ack/Pta pathways used to synthesize acetate. Ultimately, propionate synthesis yields 2 ATP, but the pathway requires multiple enzyme steps and cofactors, suggesting this overflow pathway could be kinetically limited (39). A small amount of lactate is secreted. Lactate is synthesized by lactate dehydrogenase from pyruvate, NADH, and a proton and is therefore energy consuming for the cell.

These original data were obtained using liquid chromatography technology, but since then, our ability to collect high-resolution untargeted one-dimensional proton nuclear magnetic resonance (1D ^1^H NMR) data and the statistical methods to deconvolute complicated spectra has evolved considerably, making untargeted NMR metabolomics of *B. theta* cultures feasible (40–42). Our study aimed to use untargeted metabolomics, systems biology, and biological modeling techniques to revisit the metabolism of this important human symbiont to account for nutrient inputs and outputs and to gain insight into how *B. theta* responds to physiological concentrations of metabolic fermentation products that are encountered in the gut ecosystem.

## RESULTS

### Untargeted metabolomics reveals *B. theta* secretes a subset of amino acids in addition to organic acid fermentation products.

**FIG 1:**
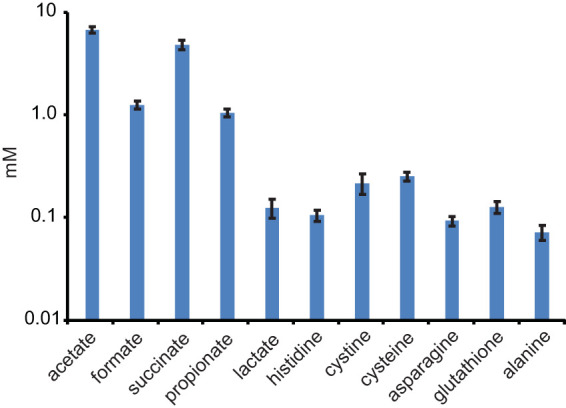
Metabolites secreted by *B. theta*. Concentrations of secreted metabolites detected after batch growth in defined minimal glucose medium (mean of 5 biological and 5 technical replicates, *n* = 25).

*B. theta* was grown in minimal defined medium on glucose as sole carbon and energy sources, and spent culture medium was analyzed using 1D ^1^H NMR to detect the secreted metabolome and to identify any new secreted metabolites (see Fig. S1 in the supplemental material). We confirmed previous observations that the major secreted metabolic products were acetate, succinate, formate, and propionate, with small amounts of lactate. In addition, we were able to detect histidine, cysteine, cystine (Cys-Cys disulfide), glutathione, asparagine, and alanine (Fig. 1).

10.1128/mSystems.00252-20.1 FIG S1 NMR metabolomics. (a) PCA scores plot generated from 1D ^1^H NMR spectra from spent culture medium (red, *n* = 25) and uninoculated medium (blue, *n* = 5). Ellipses correspond to the 95% confidence interval for a normal distribution. (b) Back-scaled loadings plot generated from a validated OPLS-discriminant analysis (DA) model (*P* value < 1 × 10^−37^) comparing the spent culture medium and uninoculated medium 1D ^1^H NMR datasets. Positive peaks indicate an increase in spent culture medium and negative peaks indicate a decrease in spent culture medium. Positive peaks labeled and added in as inlets are numbered as follows: 15, formate; 13 and 14, histidine at 7.12 and 7.96 ppm; 12, hematin; 11, glucose; 2 and 10, alanine at 1.48 and 3.83 ppm; 8, cysteine at 3.05, 3.09, and 3.99 ppm; 9, cysteine at 3.20, 3.42, and 4.11 ppm; 7, asparagine at 2.97 ppm; 6, succinate; 5, glutathione at 2.50 ppm; 4, acetate; 3, lactate at 1.33 ppm. Peaks colored red are scaled 4× and colored green are scaled 2× to their actual intensities as depicted in the full-width spectrum in blue. Download FIG S1, PDF file, 1.8 MB. Copyright © 2020 Catlett et al. 2020 Catlett et al. This content is distributed under the terms of the Creative Commons Attribution 4.0 International license.

**FIG 2:**
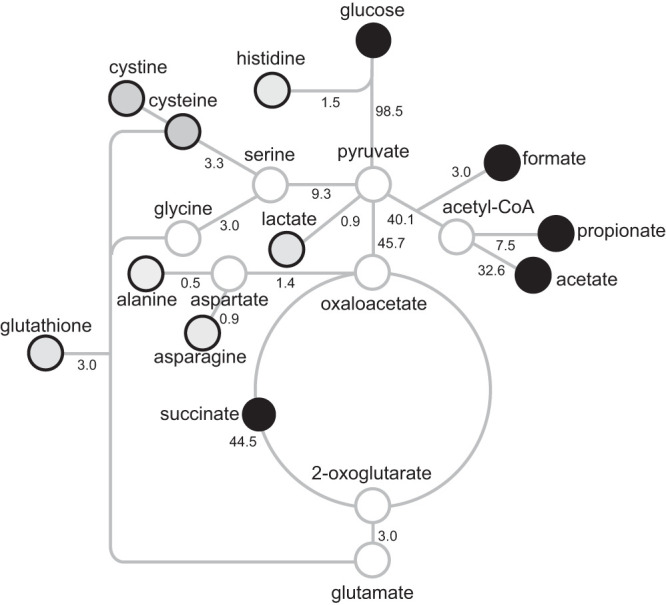
Secretion fluxes of organic acids and amino acids in defined minimal medium. Numbers represent percent mole carbon fluxes (not shown, CO_2_ inferred, 4.6%). Gray outlined circles represent undetected intracellular metabolic nodes. Black outlined circles indicate secreted metabolites. Shading is proportional to concentration in culture medium.

These endpoint metabolic products were then used to build a secretion flux map (Fig. 2). Notably, relatively few metabolites of similar size, chemical composition, reactivity, or metabolic importance to the cell were detected. Accordingly, these results suggest the secreted metabolites were products of specific cellular processes rather than through nonspecific leaky transporters.

The secretion of amino acids is significantly lower than the major organic acid fermentation products (excluding lactate) but also suggests these metabolites are overflows for purine metabolism (histidine), the TCA cycle (alanine, asparagine, and glutathione), and the serine cycle (cysteine/cystine and glutathione) (Fig. 2). Notably, the amino acid secretions were generally lower than those of lactate, suggesting amino acid secretion is less favorable, likely reflecting the fact that amino acid synthesis is energetically costly and requires multiple enzymatic steps in contrast to a single enzyme for lactate synthesis. The network map illustrates that in minimal defined medium, secreted products can be easily derived from pyruvate, acetyl-CoA, and succinate after minor biochemical transformation. This suggests the secreted metabolites are “overflow” from the EMP pathway.

### *B. theta* growth is inhibited by acetate and formate.

Acetate and formate are major metabolic products of many organisms (43), and it is thought that both acetate and formate may inhibit cell growth by feedback inhibition and/or by transporting protons across the cell membrane and collapsing the transmembrane ion gradient necessary for ATP synthesis (44, 45). This suggests that the acetate and formate produced by competing organisms in the gut may also have a strong inhibitory effect on *B. theta* metabolism. We tested these hypotheses by growing *B. theta* with increasing acetate, formate, or a combination of both in a culture medium at physiological concentrations (46).

**FIG 3:**
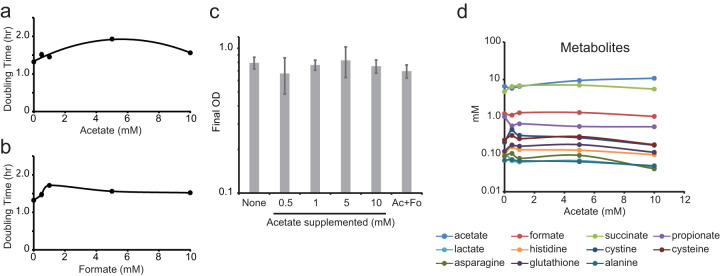
Effect of metabolic feedback inhibition on growth. (a) Population doubling time of cultures on defined minimal medium supplemented with acetate (*n* = 8 biological replicates, *P* < 0.01 versus 0 mM, *r*^2^ = 0.94). (b) Population doubling time of cultures on defined minimal medium supplemented with formate (*n* = 5 biological replicates, *P* < 0.01 versus 0 mM). (c) Final optical densities of cultures with and without supplementation of 10 mM acetate (Ac) and 10 mM formate (Fo) (*n* = 5 biological replicates, *P* > 0.05 versus 0 mM). (d) Concentrations of secreted metabolites with increasing acetate supplementation (means from 5 biological and 5 technical replicates, *n* = 25). *P* values are shown in Table S1 in the supplemental material. Curves were fit according to parabolic functions (a) or least-squares regression (b and d). Error bars may be obscured by symbols.

When *B. theta* is grown in minimal defined medium with increasing concentrations of acetate or formate, population doubling time increased by approximately 25% (Fig. 3a and b; Table 1). Conversely, the final optical density of the culture was not affected by supplementation with acetate and/or formate (Fig. 3c), and because optical density (OD) and biomass are correlated, it suggests that biomass yield is also not affected (47). These data suggest ATP synthesis and metabolic efficiency have not been altered. Instead, a direct or indirect kinetic biochemical feedback inhibition is the primary factor in acetate- and formate-dependent inhibition of *B. theta* growth. The stationary-phase cultures were observed to exhibit a modest statistically significant decrease in pH from 7.14 ± 0.064 to 7.03 ± 0.051 (*P* = 0.03) with the addition of acetate despite the medium being buffered at pH 7.2 with 1 M potassium phosphate (Table 2). The total amount of secreted organic acids and supplemental acetate cannot account for the observed pH change. Thus, the drop in pH may be attributed to an increase in CO_2_ concentration, which is converted to carbonic acid (H_2_CO_3_) with a pK_a_ of 3.6 in water. An increase in the CO_2_ partial pressure produced by cells in sealed anaerobic culture tubes is known to decrease the pH of culture medium (48).

**TABLE 1:** Effect of formate and acetate on *B. theta* growth rate in defined medium

Treatment^*a*^	Doubling time (h)	SD	*P* value	
			vs 0 mM	vs 10 mM Ac+10 mM Fo
0 mM	1.322	0.047	1	
0.5 mM Ac	1.516	0.052	0.000	0.000
1 mM Ac	1.452	0.034	0.000	0.000
5 mM Ac	1.929	0.037	0.000	0.000
10 mM Ac	1.562	0.028	0.000	0.000
0.5 mM Fo	1.474	0.042	0.000	0.000
1 mM Fo	1.718	0.078	0.000	0.704^*b*^
5 mM Fo	1.561	0.034	0.000	0.000
10 mM Fo	1.521	0.034	0.000	0.000
10 mM Ac+10 mM Fo	1.734	0.05	0.000	1

a Ac, acetate; Fo, formate. Data were obtained from six biological replicates (*n* = 6).

b Not statistically significant (*P* > 0.05).

**TABLE 2:** pH of stationary-phase cultures in buffered medium

Treatment^*a*^	pH	SD	*P* value vs 0 mM
0 mM control	7.14	0.064	1
0.5 mM Ac	7.07	0.079	0.196^*b*^
1 mM Ac	7.03	0.077	0.059^*b*^
5 mM Ac	7.06	0.034	0.064^*b*^
10 mM Ac	7.03	0.051	0.030
10 mM Ac+10 mM Fo	7.06	0.046	0.083^*b*^

a Ac, acetate; Fo, formate. Data were obtained from six biological replicates (*n* = 6).

b Not statistically significant (*P* > 0.05).

### Feedback inhibition by acetate causes suppression of metabolite secretion.

**FIG 4:**
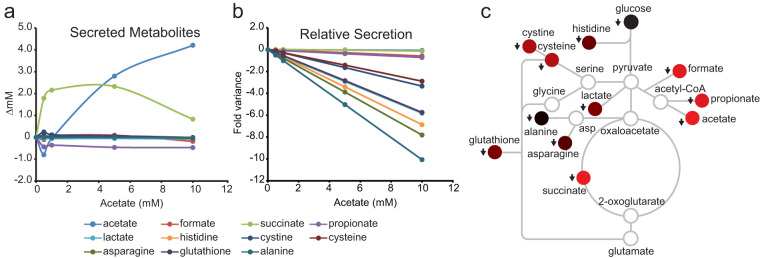
Effect of acetate feedback inhibition on secretion fluxes. (a) Concentrations of secreted metabolites with increasing acetate supplementation (means from 5 biological and 5 technical replicates, *n* = 25). *P* values are shown in Table S2. Error bars may be obscured by symbols. (b) Change in secretion fluxes with increasing acetate supplementation (means from 5 biological and 5 technical replicates, *n* = 25). *P* values are shown in Table S3. (c) Effect of acetate feedback inhibition (10 mM) mapped onto a metabolic network. Red, decreased secretion. Shading is proportional to flux magnitude. Gray outlined circles represent undetected intracellular metabolic nodes. asp, aspartate.

The concentration for each of the metabolites in the culture medium changed independently as a function of the amount of supplemental acetate (Fig. 3d). The effect of supplemental acetate on metabolite secretion (*x*_sec_) was unmasked (Fig. 4a) by subtracting the amount of each metabolite in the 0 mM control treatment (*x*_init_) from the amount of the metabolite observed (*x*_obs_) after acetate supplementation.

10.1128/mSystems.00252-20.4 TABLE S2 Student’s *t* test *P* values for metabolomics data in Fig. 4a. *P* values are given for the change in concentration of each metabolite measured in spent medium as acetate is supplemented from 0 to 10 mM. Download Table S2, DOCX file, 0.1 MB. Copyright © 2020 Catlett et al. 2020 Catlett et al. This content is distributed under the terms of the Creative Commons Attribution 4.0 International license.

10.1128/mSystems.00252-20.5 TABLE S3 Student’s *t* test *P* values for metabolomics data in Fig. 4b. Slope and Pearson correlation coefficient (*r*^2^) values are given for the change in concentration of each metabolite measured in spent medium as acetate is supplemented from 0 to 10 mM. Download Table S3, DOCX file, 0.1 MB. Copyright © 2020 Catlett et al. 2020 Catlett et al. This content is distributed under the terms of the Creative Commons Attribution 4.0 International license.

The observed concentration for each metabolite should be the same as in the initial 0 mM treatment condition if the supplemental acetate had no effect on metabolite secretion. The *x*_sec_ should also be equal to zero if the metabolite is neither a substrate nor product of acetate metabolism (null hypothesis). Instead, the concentration for each secreted metabolite changed as a result of the additional acetate in the culture medium. For instance, acetate only increased by 4.21 (± 0.55) mM in the culture medium after the 10 mM acetate treatment (Fig. 4a). This is significantly less than expected if no feedback inhibition occurred and the acetate concentrations were simply additive. The acetate concentration detected in the culture medium should have been the sum of the total amount of acetate derived from glucose (6.6 ± 0.5 mM) (Fig. 3d) plus the 10 mM acetate supplemented for a final concentration of 16.60 mM. Similar decreases in secretion were observed for formate (0.18 ± 0.02), propionate (0.47 ± 0.02), and amino acids, while succinate (0.83 ± 0.13 mM) secretion increased. These results suggest supplemented acetate was taken up by cells and altered metabolic fluxes such that secretion of acetate, formate, propionate, and amino acids are suppressed, while a portion of the acetate is secreted as succinate.

Next, the fold variance (*x*_var_) between the observed and the null model was calculated assuming the secretions of acetate and the other metabolites are correlated (Fig. 4b). The observed concentration of each metabolite indicated a negative variance from the null model. As acetate supplementation increased, the magnitude of the variance also increased, indicating “missing” metabolites. The increasing negative correlation as a function of supplemental acetate suggests these missing metabolites are a result of an unknown inhibitory mechanism or process.

The slope of the linear regression Δ*x*_var_ is the relative molar acetate suppression coefficient (Table 3). Accordingly, alanine and the amino acids have the highest molar acetate suppression coefficient despite acetate secretion having a higher magnitude of inhibition. Relative molar flux suppression was mapped onto a metabolic network as shown in Fig. 4c. The flux analysis shows that acetate supplementation has the highest inhibitory effect on alanine secretion, an intermediate effect on amino acid secretion, and the smallest inhibitory effect on the major fermentation products: acetate, succinate, formate, and propionate.

**TABLE 3:** Acetate suppression coefficients of secreted metabolites

Metabolite	Fold suppression coefficient (Δ*x*_var)_	Pearson correlation (*R*^2^)
Acetate	−0.00535	0.93736
Formate	−0.05783	0.99941
Succinate	−0.01478	0.94946
Propionate	−0.07022	0.99859
Cysteine	−0.28908	0.99990
Cystine	−0.33741	0.99955
Glutathione	−0.57522	0.99995
Lactate	−0.57985	0.99998
Histidine	−0.68781	0.99997
Asparagine	−0.78083	0.99999
Alanine	−1.00662	1.00000

### Feedback inhibition is the primary driver of acetate inhibition effects.

In addition to a direct competitive feedback inhibition (e.g., carbonic anhydrase) (49), supplemental acetate and formate may also induce a noncompetitive inhibition through posttranslational modification (formylation) (50). A direct or indirect effect on gene expression may occur as growing cells adapt to the stress. To determine the relative contribution of feedback inhibition and gene expression changes to secretion fluxes, the following hypotheses were modeled with the assumption that secreted metabolites mirror intracellular metabolic fluxes: (a) secretion is additive with no destruction and no regulation (see Fig. S2a); (b) secretion fluxes are constant with balanced secretion and absorption (Fig. S2b); (c) there is feedback inhibition with no regulation (Fig. S2c); (d) there is synergistic negative feedback inhibition with compensatory gene regulation (Fig. S2d); and (e) there is positive upregulation in response to increasing acetate (Fig. S2e). The modeling results (Fig. 4b versus Fig. S2d) indicate that the experimental data most closely match a feedback inhibition model with no gene regulation. The only exception is for acetate and succinate, which were discussed previously. *B. theta* appears to respond to low concentrations of exogenous acetate (<0.5 mM) by adjusting gene expression to decrease metabolic flux, but at acetate levels up to 10 mM, no further changes in gene expression or other metabolic rerouting occur.

10.1128/mSystems.00252-20.2FIG S2Theoretical modeling of the effect of acetate supplementation on the observed secreted metabolites. Models assume a fraction of supplemented acetate is converted to secreted metabolite *x*. $x_{\sec}=x_{\mathrm{init}}+aA_{\sup}+bA_{\sup}$. (a) Secretion is additive with no destruction and no regulation when a>0 and b=0. *x* axis, arbitrary time units; *y* axis, Δ*x*_sec_, arbitrary change in concentration of metabolite *x* detected in the culture medium. Colors represent concentrations of supplemented acetate. (b) Secretion fluxes are constant with balanced secretion and absorption when $aA_{\sup}+bA_{\sup}=\;-x_{\mathrm{init}}$. *x* axis, arbitrary time units; *y* axis, Δ*x*_sec_, arbitrary change in concentration of metabolite *x* detected in the culture medium. Colors represent concentrations of supplemented acetate. Symbols and lines overlap and may not be visible. (c) Feedback inhibition with no regulation when a<0 and b=0. *x* axis, arbitrary time units; *y* axis, Δ*x*_sec_, arbitrary change in concentration of metabolite *x* detected in the culture medium. Colors represent concentration of supplemented acetate. (d) Synergistic negative feedback inhibition with compensatory gene regulation when a<0 and b<0. *x* axis, arbitrary time units; *y* axis, Δ*x*_sec_, arbitrary change in concentration of metabolite *x* detected in the culture medium. Colors represent concentrations of supplemented acetate. Symbols and lines overlap and may not be visible. (e) Positive upregulation in response to increasing acetate when a>0 and b>0. *x* axis, arbitrary time units; *y* axis, Δ*x*_sec_, arbitrary change in concentration of metabolite *x* detected in the culture medium. Colors represent concentrations of supplemented acetate. Symbols and lines overlap and may not be visible. Download FIG S2, EPS file, 1.0 MB.Copyright © 2020 Catlett et al.2020Catlett et al.This content is distributed under the terms of the Creative Commons Attribution 4.0 International license.

### Formate causes synergistic feedback inhibition of acetate, propionate, succinate, histidine, cysteine, and glutathione.

**FIG 5:**
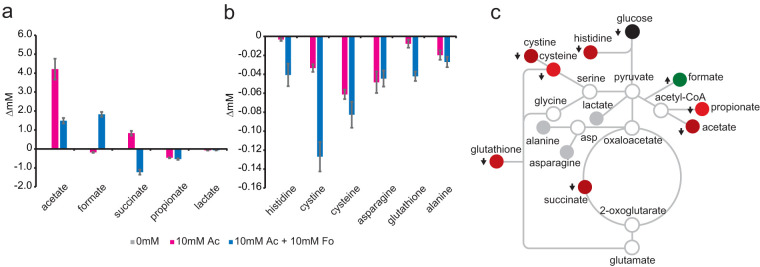
Effect of formate supplementation on acetate feedback inhibition. (a) Change in organic acid secretion with and without supplementation of 10 mM acetate or a combination of 10 mM acetate (Ac) and 10 mM formate (Fo). (b) Change in secreted amino acids with and without supplementation. (c) Effect of 10 mM formate supplementation on 10 mM acetate feedback inhibition mapped to the metabolic network. Green, increased secretion; red, decreased secretion; gray, no significant difference between 10 mM acetate versus 10 mM acetate and 10 mM formate conditions. Shading is proportional to flux magnitude. Gray outlined circles represent undetected intracellular metabolic nodes. asp, aspartate. *P* values for data in panels a and b are shown in Table S4. Error bars may be too small to see.

Formate is a major fermentation by-product encountered in anaerobic environments. Like acetate, formate is produced from the Embden-Meyerhof-Parnas fermentation pathway; however, formate can be used as either an energy sink or an energy source depending on the levels of CO_2_ and H_2_ or the redox state of ferredoxin. Thus, high formate concentrations may be synergistic, additive, or have independent effects on secretion fluxes compared to those for acetate alone. To differentiate between these possibilities, cultures were supplemented with 10 mM both acetate and formate. NMR metabolomics was then used to characterize the secreted metabolome. Growth experiments demonstrated that formate had a synergistic effect on population doubling time (Table 1). Supplemented acetate and formate appeared to be taken up from the culture medium, but the additional acetate and/or formate did not affect biomass yield or pH. No new secreted metabolites were observed in the culture medium, but a subset of organic acids (Fig. 5a) and amino acids (Fig. 5b) did exhibit concentration changes relative to those in treatments with only 10 mM acetate. Notably, lactate, alanine, and asparagine secretion levels were unchanged. These data suggest those biosynthetic reactions are unaffected by acetate or formate, while other reactions are either directly affected by enzyme inhibition or indirectly affected by changes in metabolic flux.

10.1128/mSystems.00252-20.6 TABLE S4 Student’s *t* test *P* values for metabolomics data in Fig. 5a and b. *P* values are given for the change in concentration of each metabolite measured in spent medium in the absence and presence of 10 mM acetate and 10 mM formate supplementation. Download Table S4, DOCX file, 0.1 MB. Copyright © 2020 Catlett et al. 2020 Catlett et al. This content is distributed under the terms of the Creative Commons Attribution 4.0 International license.

### Visualization of dynamic metabolite secretion and effects of feedback inhibition.

Animations of the dynamics of metabolic secretion (Movie S1), acetate inhibition (Movie S2), and the modulation of acetate inhibition by formate supplementation (Movie S3) are presented in the supplemental material. The animated models assume linear secretion fluxes and illustrate the accumulation of secreted products in the culture medium as a function of time and acetate and/or formate supplementation.

10.1128/mSystems.00252-20.7 MOVIE S1 Animated secretion fluxes of organic acids and amino acids in defined minimal medium. Numbers represent percent mole carbon fluxes (not shown, CO_2_ inferred, 4.6%). Gray outlined circles represent undetected intracellular metabolic nodes. Black outlined circles indicate secreted metabolites. Shading is proportional to concentration in culture medium from 0% (red circles) to yellow (50%) to green (100%) carbon flux. Download Movie S1, GIF file, 0.1 MB. Copyright © 2020 Catlett et al. 2020 Catlett et al. This content is distributed under the terms of the Creative Commons Attribution 4.0 International license.

10.1128/mSystems.00252-20.8 MOVIE S2 Animated effect of acetate feedback inhibition on secretion fluxes. The effect of acetate feedback inhibition (10 mM) mapped onto a metabolic network. Gray outlined circles represent undetected intracellular metabolic nodes. Black outlined circles indicate secreted metabolites. Shading is proportional to change in carbon flux compared to no acetate control from 0% (red circles) to yellow (50%) to green (100%). Download Movie S2, GIF file, 0.1 MB. Copyright © 2020 Catlett et al. 2020 Catlett et al. This content is distributed under the terms of the Creative Commons Attribution 4.0 International license.

10.1128/mSystems.00252-20.9 MOVIE S3 Animated effect of formate supplementation on acetate feedback inhibition. The effect of formate (10 mM) and acetate feedback inhibition (10 mM) mapped onto a metabolic network. Gray outlined circles represent undetected intracellular metabolic nodes. Black outlined circles indicate secreted metabolites. Shading is proportional to concentration in culture medium from 0% (red circles) to yellow (50%) to green (100%) carbon flux. Download Movie S3, GIF file, 0.1 MB. Copyright © 2020 Catlett et al. 2020 Catlett et al. This content is distributed under the terms of the Creative Commons Attribution 4.0 International license.

### High-glucose models do not predict feedback inhibition by acetate or formate.

Empirical modeling suggested that the bioenergetics of glucose metabolism does not change when acetate and/or formate accumulates in the culture medium. Therefore, the observed changes in secreted metabolites must be due to increased CO_2_ and/or H_2_ secretion fluxes. To determine if *in silico B. theta* metabolic models can reproduce the observed growth phenotypes, we conducted a series of *in silico* experiments to obtain flux values for all reactions in the metabolic model and exchange fluxes for all substrate compounds and product metabolites.

**FIG 6:**
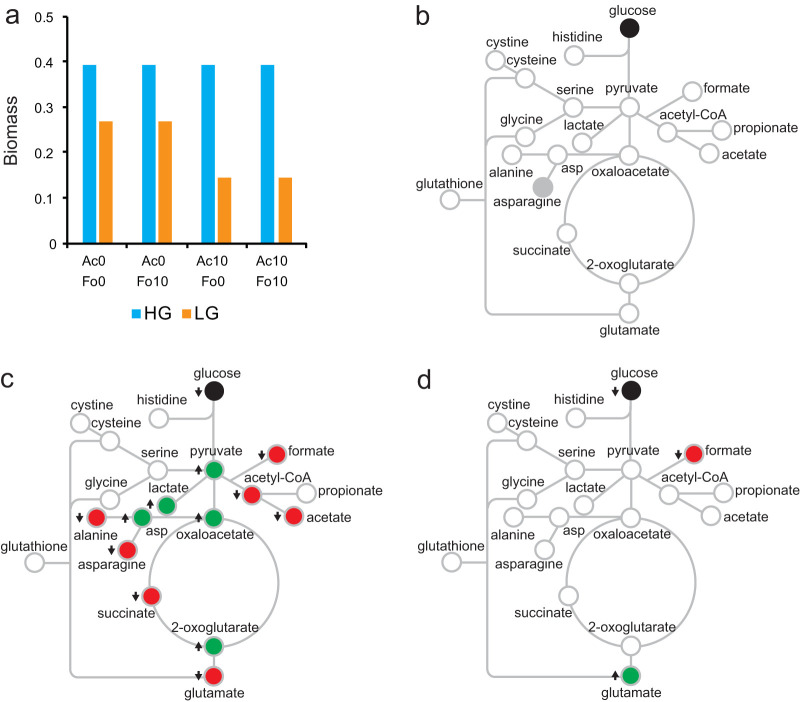
Simulation of the effect of feedback inhibition on metabolism. (a) Predicted biomass in high-glucose (HG) and low-glucose (LG) medium as acetate (Ac) and formate (Fo) concentrations were varied from 0 mM to 10 mM. (b) Predicted effect of acetate and/or formate on exchange fluxes in HG medium. (c) Predicted effect of acetate on exchange fluxes in LG medium. (d) Predicted effect of formate on exchange fluxes in LG medium. Green, increased exchange flux; red, decreased exchange flux; gray, no net change in exchange flux due to metabolic rerouting; white, no change predicted.

A limited effect on the key reaction fluxes (defined in millimoles per gram cell dry weight per hour) was observed under high-glucose (0.02% [wt/vol]) conditions. In the high-glucose flux balance analysis (FBA) models, there were no changes in predicted biomass, which remained constant at 0.39 in all four experiments (Fig. 6a), or in exchange fluxes with either acetate and/or formate supplementation (Fig. 6b). There was only one discrepancy in a predicted reaction flux. The model shifts from using asparagine synthetase (aspartate + glutamine + ATP + H_2_O ↔ asparagine + glutamate + AMP + PP_i_) to using aspartate ammonium ligase (aspartate + NH_3_ + ATP → asparagine + AMP + PP_i_) when either acetate and/or formate is included in the culture medium (see Data Set S1). However, the glutamate, glutamine, asparagine, and aspartate exchange fluxes were not altered in the model despite the changes in reaction flux. We confirmed that either pathway can be deleted in the model and results in the same exchange fluxes regardless of whether acetate or formate is supplemented. Thus, the simulations show either metabolic pathway for asparagine synthesis can occur interchangeably in *B. theta* under the culture conditions we modeled.

10.1128/mSystems.00252-20.10 DATA SET S1 Model parameters and output flux data for high-glucose and low-glucose FBA models are included in an Excel spreadsheet. Download Data Set S1, XLSX file, 0.1 MB. Copyright © 2020 Catlett et al. 2020 Catlett et al. This content is distributed under the terms of the Creative Commons Attribution 4.0 International license.

### Modeling suggests acetate and formate affect metabolism when glucose concentrations are low.

The presence of acetate under low-glucose conditions (0.002% [wt/vol]) results in an abundance of metabolic changes. When acetate was added, whether in the presence or absence of formate, biomass decreased to 0.14 (Fig. 6a). Exchange flux values for several metabolites were also affected (Fig. 6c and Data Set S1). Some pathways, such as malate dehydrogenase, serine ammonia-lyase, and formate-tetrahydrofuran (THF) ligase show a net flux of zero. Other reaction fluxes, such as those corresponding to enzymes lactate dehydrogenase, succinyl-CoA synthetase, and aspartate aminotransferase, incurred a change in directionality. The reaction for aspartate oxidase had a zero-net flux under glucose-only or glucose and formate conditions; however, the reaction had a flux of 0.0009 when acetate was present. Other reactions retained their directionality, but the net flux exhibited a change in magnitude. In the forward direction, examples of reactions that increased exchange flux in the presence of added acetate included pyruvate kinase and aspartate aminotransferase, while pyruvate carboxylase, pyruvate synthase, pyruvate dehydrogenase, and acetate kinase had decreased flux. In the reverse reaction direction, pyruvate phosphate dikinase had increased exchange flux and phosphotransacetylase had decreased flux. No additional effect was observed when formate was combined with acetate.

The interchangeability of asparagine synthetase and aspartate ammonia ligase pathways for asparagine synthesis was also observed under low-glucose conditions, similar to what was seen under high glucose conditions (Fig. 6d). In addition, when formate was added under low glucose conditions, the net flux of the pyruvate formate-lyase reaction (formate + acetyl-CoA ↔ CoA + pyruvate) decreased from −3.74 to −0.122, where a negative flux value indicates the reaction was being executed from right to left. Under this condition, biomass decreased to a value of 0.27 (Fig. 6a).

The low-glucose model also predicts increased uptake of acetate and decreased uptake of CO_2_ and cysteine when acetate is supplemented into the culture medium, which is consistent with metabolomics data and the observed pH decrease. None of the simulations resulted in changes in the secretion of organic acids or amino acids to the culture medium. Modeling instead showed decreased production of a small amount of molecular oxygen, lack of nitrite secretion, and increased secretion of xanthine under low-glucose conditions with addition of acetate.

## DISCUSSION

### Feedback inhibition reveals metabolic plasticity and resiliency of *B. theta*.

*B. theta* is a ubiquitous and abundant member of the human gut microbiome. Accordingly, *B. theta* is an attractive organism for investigating the interaction between genes, environment, and system-level behaviors. *B. theta* is a strict anaerobe grown in sealed culture tubes. Thus, by the law of conservation of mass, all mass inputs (culture medium ingredients) and outputs (biomass and secreted metabolites) are accounted for in the cell culture. Secreted metabolites, especially organic acids and amino acids, are important mediators in microbial food webs and may play simultaneous roles as nutrients, stimulators, and inhibitors. In this manner, secreted metabolites may affect overall system behavior.

Metabolomics and cell growth data suggest fermentation products, acetate and formate, cause large metabolic changes even when biomass yield is unaffected. *B. theta* has two bidirectional acetate enzyme systems, Ack/Pta and acetyl-CoA pathways, and the metabolomics and modeling data are consistent with *B. theta* using both of these pathways to secrete acetate as an “overflow” of acetyl-CoA biosynthesis. Overflow metabolism has been studied extensively in E. coli (37), where it is thought that excess carbon from glucose is secreted as acetate due to metabolic bottleneck at pyruvate and acetyl-CoA as a result of redox imbalance (38). In E. coli and *Salmonella*, secreted acetate is recouped by the Ack and Pta enzymes during late stationary phase (51), where the glyoxylate shunt is used to incorporate acetyl-CoA into biomass (52). While the enzymes involved in acetate secretion and uptake are conserved between E. coli and *B. theta*, *B. theta* lacks the glyoxylate shunt and is an anaerobe that cannot carry out oxidative respiration. The addition of acetate to the culture medium caused the inhibition of acetate, formate, and propionate secretion by *B. theta*, but increased succinate secretion as the next available “overflow valve.” This result may be explained by feedback inhibition of acetyl-CoA hydrolysis and increased succinyl-CoA hydrolysis later in the TCA cycle. Consequently, there was a decrease in the secretion of other metabolic products (cysteine and other amino acids) downstream of succinate biosynthesis. *B. theta* biomass yield was unaffected by supplemented acetate, as expected by the lack of a glyoxylate shunt. The biomass yield did not increase despite a decrease in the secretion of amino acids, indicating intracellular amino acid biosynthesis was sufficient for maximum growth.

The addition of formate to the culture medium caused an inhibition in the secretion of acetate and a subset of amino acids, while metabolites derived from oxaloacetate and aspartate (lactate, asparagine, and alanine) were not affected. Formate and acetate are both synthesized from pyruvate but have different effects on downstream “overflow” metabolites. One possible explanation for this difference is the fact that formate is a substrate for C_1_ metabolism (ultimately for glycine, serine, cysteine, and methyl transfer reactions). Thus, the addition of formate increases the synthesis of amino acids, lactate, and pyruvate (through serine ammonia-lyase), which may compensate for the inhibition of acetate, succinate, and propionate.

Unexpectedly, not all metabolites were secreted and no new metabolites were detected in culture medium as a result of acetate and/or formate inhibition. Several central metabolites are simply too large to be nonspecifically secreted (phosphosugars and CoA-oxoacids), but many nonsecreted TCA and amino acid biosynthetic intermediates are chemically similar to secreted metabolites. This suggests that metabolite secretion is highly discriminated by transporters. *B. theta* also seems unable to relax transporter specificity or to produce new transporters through changes in gene expression. In effect, *B. theta* did not relieve acetate or formate inhibition by secreting other biosynthetic intermediates.

Another surprise was the observation that biomass yield was unaffected by the physiological concentrations of acetate and/or formate. Accordingly, net bioenergetics (ATP moles synthesized per mole substrate consumed) were also likely unaffected, even though the rate of growth was significantly lower and there were large changes in secretion profiles. The FBA model was able to accurately predict that acetate and formate supplementation does not affect biomass (Fig. 3c versus Fig. 6a) under high-glucose conditions. This leads to the question of where the unaccounted carbon could have gone. A likely explanation is that the missing carbon mass was released as CO_2_. Increased CO_2_ synthesis would manifest as a decrease in pH but would not necessarily affect CO_2_-yielding decarboxylation reactions. At the partial pressures tested here, decarboxylation reactions are virtually irreversible unidirectional reactions. Malate dehydrogenase reversibly catalyzes the decarboxylation of oxaloacetate to produce pyruvate and CO_2._ Malate dehydrogenase also reversibly oxidizes malate to produce pyruvate, CO_2_, and NADPH. Our results can be explained if the forward malate dehydrogenase reaction is favored, with increased pyruvate being secreted as acetate and formate. The low-glucose FBA models support this hypothesis and show that addition of acetate results in nearly a 50% decrease in CO_2_ uptake from the culture medium (see Data Set S1 in the supplemental material).

The FBA models for *B. theta* were unable to model secretion of organic acids and amino acids (Fig. 2 versus Data Set S1), and though lowering the glucose concentration in the growth medium caused some metabolic network changes, they did not completely predict the effect of acetate and formate on exchange fluxes (compare Fig. 4c versus Fig. 6c and Fig. 5c versus Fig. 6d). One possibility is that the carbon predicted to be secreted as xanthine (which was not detected experimentally) is instead used to synthesize organic acids and amino acids. Under both the high-glucose and low-glucose conditions, there were also unexpected results with respect to nitrite and ammonia fluxes, suggesting unexplored C/N metabolic or regulatory relationships in *B. theta*. Another possibility is that transporters (either specifically or nonspecifically) secrete accumulated metabolite pools as part of “overflow metabolism.” These discrepancies likely reflect technological limitations of *in silico* modeling, such as an inability to predict allosteric or competitive inhibition, gene expression changes that might result in specific or nonspecific activity of transmembrane transporters, or perhaps the activity of poorly characterized enzymes or nonspecific aminotransferases and decarboxylases (or other enzymes) that may affect exchange fluxes in unknown ways. By using untargeted NMR metabolomics, we were able to detect and quantify metabolites in culture medium with minimal sample processing in a relatively “agnostic” approach. NMR data sets can be used to produce secretion flux maps that describe metabolic behaviors without requiring genomic, biochemical, or transcriptomic information or, in the reverse direction, may be used to infer the existence of unknown biochemical pathways. We suggest that untargeted NMR metabolomics may be a useful tool to inexpensively curate genome-scale metabolic models and could be essential for developing accurate dynamic FBA models.

### Are *B. theta* secretion signals of relevance to a host-microbiome system?

It has been hypothesized that amino acids can function as a “shared good” in microbe-host ecosystems in which secreted “overflow” amino acids can be taken up by the host or neighboring community members (commensalism). In this regard, a cell can dispose of its excess amino acids while also benefitting near neighbors (mutualism). It is also possible that these secreted amino acids are used in mutually beneficial metabolite exchange (syntrophy) (53–55), for example, when metabolite secretion causes metabolic feedback inhibition that can be relieved by a consumer partner. Detection of amino acid products in *B. theta* culture supernatants supports the postulation that *B. theta* is primed to participate in such cross-feeding interactions in the gut (56). Humans are known to require branched-chain amino acids (leucine, isoleucine, and valine) and conditionally essential amino acids (arginine, proline, cysteine, and glycine) as well as lysine, threonine, methionine, tryptophan, phenylalanine, and histidine (57). The essential amino acid histidine and the conditionally essential amino acids cysteine and glycine (as glutathione) were observed to be secreted by *B. theta*.

Besides nutrition, amino acids also have a wide range of roles in gut epithelial metabolism and gut immune/neurological function. In fact, several amino acids are secreted at high concentrations by *B. theta*. In gut epithelial cells, glutamate, aspartate, and glutamine are substrates for ATP synthesis, glutamine, glycine, and aspartate are used for nucleic acid synthesis, and threonine, cysteine, and proline are used for mucin synthesis. Thus, a symbiotic relationship may exist between *B. theta* and gut epithelium, where *B. theta* may provide essential amino acids critical for gut epithelial metabolism.

Glutathione is a tripeptide of cysteine, glutamate, and glycine, which also has an important role in epithelial cell viability. It can provide a source of amino acids, it can protect against toxic xenobiotics, and it is important for cell signaling. Glutathione also serves as a redox buffer and can protect cells from reactive oxygen species (ROS) or oxidative stress (58). Thus, it is notable that *B. theta* was observed to secrete 121 ± 16 μM glutathione into the culture medium. Since lactic acid bacteria produce H_2_O_2_ in the gut to compete with anaerobes such as *B. theta* for glycan nutrients (59, 60), the secretion of glutathione by *B. theta* may protect *B. theta* from these competing microbes. Secreted glutathione may also protect *B. theta* from oxidative stress generated by host epithelia at the microbe-host interface (61–63). The amino acid components of glutathione, glutamine, and glycine may act as neurotransmitters between gut epithelia and the nerve cells that innervate the intestinal tract.

Cysteine (242 ± 22 μM) and cystine (209 ± 47 μM) were also secreted by *B. theta*. Cysteine and cystine, like glutathione, can abiotically react with ROS or xenobiotic compounds to protect cells from oxidative damage. Histidine was also secreted at high levels (101 ± 12 μM), which was nearly equivalent to that of lactate (120 ± 25 μM). Histidine is an essential amino acid and is a precursor to the immunological effector histamine. Secretion of amino acids and glutathione by *B. theta* could potentially play an important role in host nutrition, oxidative stress, neurological function, and immunology.

Our metabolomics, theoretical modeling, and cell viability results support the hypothesis that microbes in complex communities modulate *B. theta*’s metabolic efficiency, which leads to changes in secreted metabolites that, in turn, are sensed as chemical messages by the microbial community and host (54). Metabolic feedback inhibition by fermentation products such as acetate and formate would be expected to function through generalized cellular processes rather than through specific quorum sensing. However, because acetate and formate are highly conserved major metabolic end products synthesized by anaerobic microbes in the millimolar and high micromolar concentration ranges, the local concentration achieved in gut microenvironments could be sufficiently high to profoundly affect metabolism of neighboring microbes and thus metabolism of the gut community as a whole.

## MATERIALS AND METHODS

### Strains and culture conditions.

Bacteroides thetaiotaomicron vpi-5482 (ATCC 29148, Buan lab strain collection number NB203) was grown in minimal defined medium as described but with minor modifications (64–66). Cultures were grown under strict anaerobic conditions at 37°C in 18-mm by 150-mm Balch tubes in either tryptone and yeast extract (TYG) growth medium (vitamin K omitted) or a defined medium (vitamin K omitted). Media were supplemented with glucose to 0.05% (wt/vol) (2.78 mM) under a 5% H_2_, 20% CO_2_, N_2_ atmosphere with the following additions as appropriate: sodium acetate (10 mM), sodium formate (10 mM), or a combination of both 10 mM sodium acetate and 10 mM sodium formate. Growth was measured using optical density at 600 nm using a Spectronic D spectrophotometer (Thermo Fisher Scientific) fitted with a Balch tube (18 mm) sample chamber. Biomass and optical density were found correlate linearly with 0.54 ± 0.056 g dry weight OD^−1^ liter^−1^ in defined medium.

### NMR sample preparation.

Five replicates of *B. theta* cultures were grown to late exponential stage in 10 ml defined medium with 0.05% glucose and one of the following concentrations of acetate: 0.5 mM, 1 mM, 5 mM, 10 mM, or a 10 mM formate and 10 mM acetate control. Cells were separated from medium with 0.2-μm filters by vacuum. Samples of filtered medium were flash frozen in liquid nitrogen and then lyophilized overnight.

### NMR data collection and analysis.

One-dimensional (1D) ^1^H NMR data collection and analysis were completed as described previously (40–42, 67–69). Briefly, samples from each class were prepared for NMR analysis by dissolving the lyophilized culture medium into 600 μl of 50 mM phosphate buffer (pH 7.2, uncorrected) in 99.8% D_2_O with 50 μM 3-(tetramethylsilane)propionic acid-2,2,3,3-d4 (TMSP). NMR spectra were recorded at 298 K on a Bruker Avance III-HD 700 MHz spectrometer equipped with a 5-mm inverse quadruple-resonance (^1^H, ^13^C, ^15^N, and ^31^P) cryoprobe with cooled ^1^H and ^13^C channels and a *z*-axis gradient. A SampleJet automated sample changer with Bruker ICON-NMR software was used to automate the NMR data collection. 1D ^1^H spectra were collected using excitation sculpting to remove the solvent signal and avoid any need for baseline corrections (70). A total of 16,000 data points with a spectral width of 5482.5 Hz, 8 dummy scans, and 128 scans were used to obtain each spectrum.

The 1D ^1^H NMR spectra were processed and analyzed using our MVAPACK metabolomics toolkit (http://bionmr.unl.edu/mvapack.php) (71). The 1D ^1^H NMR spectra were Fourier transformed and phased prior to normalization using phase scatter correction (72). Residual solvent peaks and noise regions were removed, and the spectra were referenced to TMSP at 0.0 ppm. The spectra were then binned using an intelligent adaptive binning algorithm (73) or aligned with the icoshift algorithm (74). The data were scaled using the Pareto method prior to principal-component analysis (PCA) or orthogonal projections to latent structures (OPLS) analysis (69).

Binned data were used for the PCA model, whereas the full spectral data were utilized for the OPLS models. OPLS model results were validated using analysis of variance of the cross-validated residuals (CV-ANOVA) significance testing (75). Fractions of explained variation (*R*^2^*_X_* and *R*^2^*_Y_*) were computed during the OPLS model training. The OPLS models were also internally cross-validated using 7-fold Monte Carlo cross-validation to compute *Q*^2^ values (76, 77).

The validated OPLS models enabled the generation of back-scaled loading plots to identify the spectral features (NMR peaks) that primarily contributed to the observed group separation. The relative peak intensities in these “pseudospectra” highlight the magnitude of the metabolite’s contribution to the group separation in the OPLS scores plot. Similarly, the relative sign of the peak indicates if the metabolite’s concentration increases or decreases due to the effects of the growth medium. All nonoverlapping ^1^H NMR peaks identified by the back-scaled loading plots as a major contributor to group separation in the OPLS scores plot were assigned to a metabolite using the Chenomx NMR suite 7.0 (Chenomx Inc., Edmonton, AB, Canada). ^1^H NMR peaks with significant overlap and multiple metabolite assignments were excluded from further analysis.

### Empirical modeling of metabolomics data.

Secretion flux maps were generated using the following equation:

(1) $$F\left(x\right)=100\text{ ∗ }\frac{\mathrm{xmol}C_{x}}{\sum_{n=1}^{i}x_{n}\mathrm{mol}C_{x_{n}}}$$

where the secretion flux (F) of any metabolite (x [mM]) is expressed as a % C mol fraction ($\mathrm{mol}C_{x}$) of the total carbon secreted.

Feedback inhibition was estimated using the following equation:

(2) $$x_{\text{sec}}=x_{\text{obs}}-x_{\text{init}}$$

where metabolite secretion (*x*_sec_) is determined by subtracting the amount of each metabolite in the 0 mM control treatment (*x*_init_) from the amount of the metabolite observed (*x*_obs_) following the addition of acetate.

The fold variance in metabolite secretion was estimated by

(3) $$x_{\text{var}}=(\frac{x_{\text{obs}}-A_{\text{sup}}}{x_{\text{init}}})-1$$

Where *x*_obs_ in the observed concentration of each metabolite, *A*_sup_ indicated the concentration of acetate supplemented, and *x*_var_ is the magnitude of each “missing” metabolite.

### *In silico* modeling and software.

*In silico* experimentation is conducted using the Department of Energy’s Systems Biology Knowledgebase (KBase) (78). A public narrative with all experiments recreated can be found in KBase (https://narrative.kbase.us/narrative/ws.53087.obj.1). Applications used are part of the fba_tools module version 1.7.6 (78). Model creation begins with the genome Bacteroides thetaiotaomicron VPI-5482 uploaded through KBase’s public NCBI RefSeq genome database. Using the “build metabolic model” application, the draft metabolic models were created from an annotated genome. The fba_tools default parameters were used. The *in silico* experimentation process with KBase consists of four steps: (i) creating a draft metabolic model from the *B. theta* genome, (ii) defining the medium composition, (iii) gap filling the draft model to add in missing reactions, and (iv) running a flux balance analysis (FBA). FBA provides a measurement of growth resulting from flux through the biomass reaction (grams of dry weight of biomass) (https://kbase.us/metabolic-modeling-faq/).

It is critical to note that the draft *B. theta* model we employed may have missing reactions (gaps) due to incorrect or incomplete functional genome annotations. We used the “gapfill metabolic model” application on the draft model to identify a minimal set of biochemical reactions that, when added to the draft model, allow it to achieve biomass on the specified media (https://kbase.us/metabolic-modeling-faq/) (79). Gap filling uses linear programming to find the optimized metabolic model that uses the fewest added reactions to satisfy the biomass reaction and to balance the flux balance equation. We gap filled once for each of the eight media in our experimentation, creating eight metabolic models. Then, starting with a base medium file containing 25 substrate compounds (full medium compositions can be found in supplemental material), we added glucose, formate, and acetate at their desired maximum uptake concentrations.

The “run flux balance analysis” (FBA) application was used to run the simulation. The FBA algorithm is a constraint-based approach that estimates growth-optimal fluxes through all the reactions specified by the metabolic network constructed in the previous step (https://kbase.us/metabolic-modeling-faq/). This resulted in a rate of biomass production as a measure of growth. For each FBA, we used the gap-filled model on the medium of interest and used each medium as input to the FBA algorithm to maximize biomass (bio1). From the output of the FBA application, the objective value was used as a measurement of growth and to capture the reaction and exchange fluxes, which were used to find flux values of interest. All data and results are presented in our public KBase narrative (https://narrative.kbase.us/narrative/ws.53087.obj.1).

The FBA models were created with either high or low levels of glucose, which were combined with either the absence or presence of acetate and/or formate. This resulted in eight experiments: high glucose, high glucose with acetate, high glucose with formate, high glucose with acetate and formate, low glucose, low glucose with acetate, low glucose with formate, and low glucose with acetate and formate. The amount of each compound used in the model is specified in the medium file, which defines maximum uptake as measured in millimoles per gram cell dry weight per hour. Low glucose was defined as 0.1 maximum uptake, high glucose was defined as 2.78 maximum uptake, and the presence of formate or acetate was set to 10 maximum uptake. The absence of formate or acetate was set to 0 maximum uptake.
