# Supplementary figures and images for: Metabolic Feedback Inhibition Influences Metabolite Secretion by the Human Gut Symbiont Bacteroides thetaiotaomicron
Source: mSystems. 2020 Sep 1;5(5):e00252-20. doi: 10.1128/mSystems.00252-20 (PMC7470985; doi:10.1128/mSystems.00252-20)

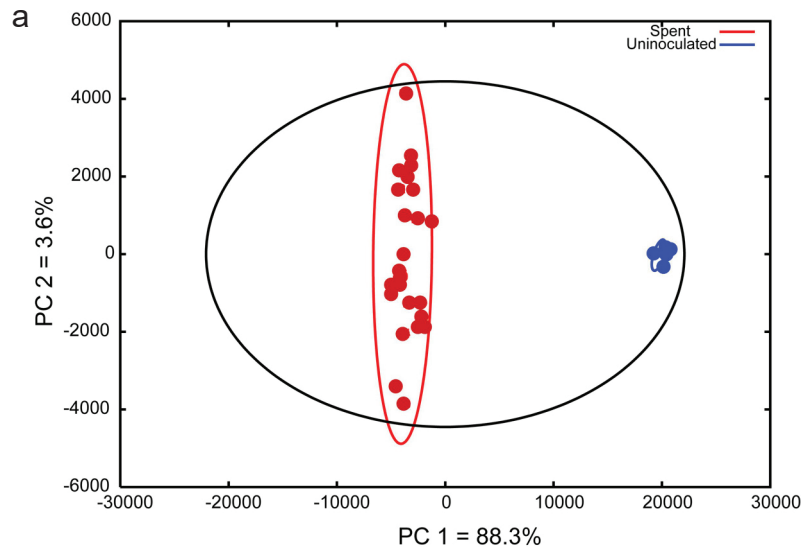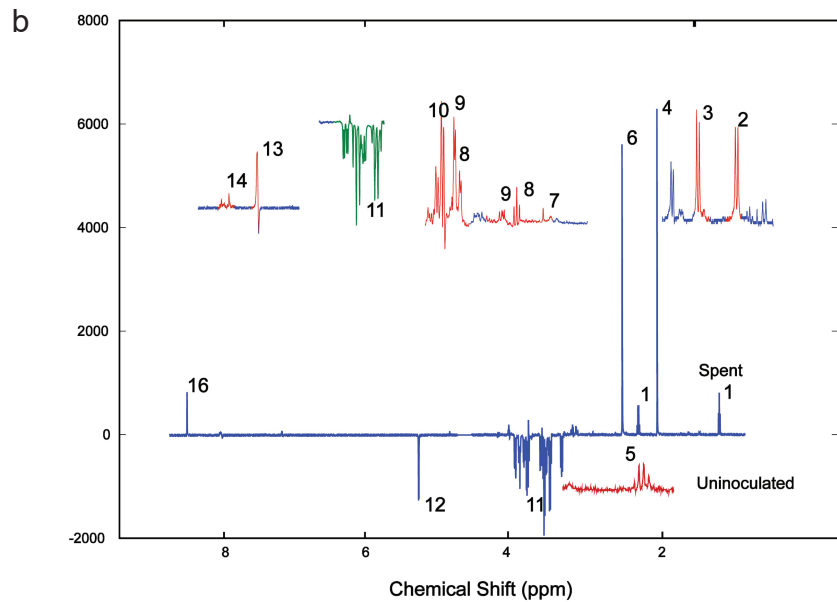

Supplement: FIG S1 [file mSystems.00252-20-sf001.pdf]

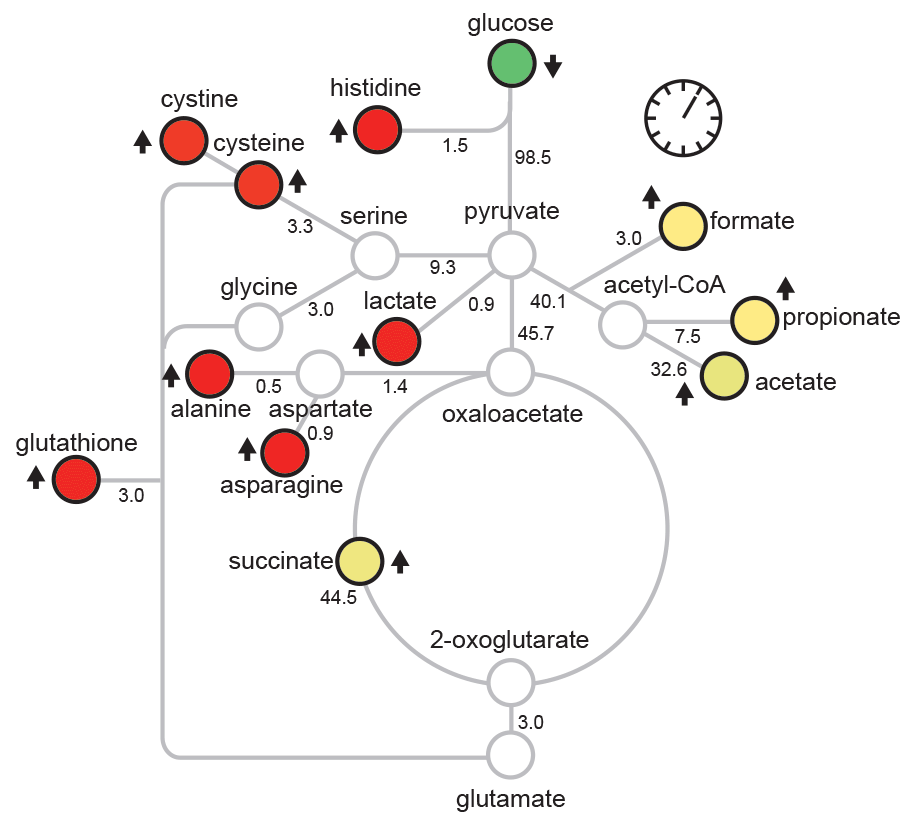

Supplement: MOVIE S1 [file mSystems.00252-20-sm001.gif]

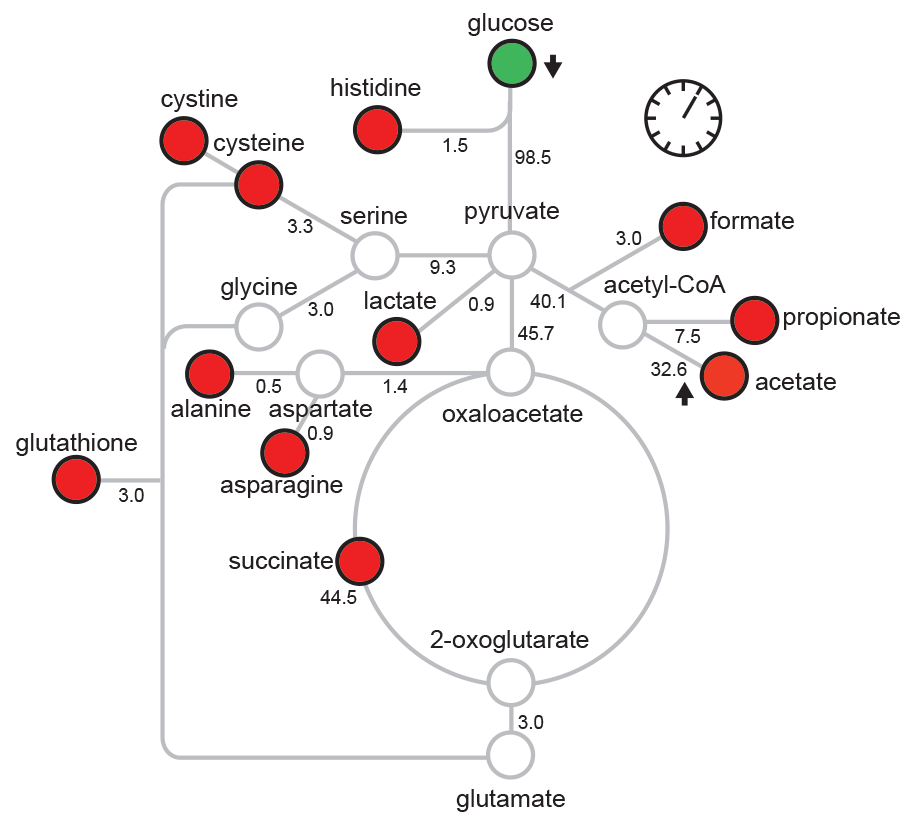

Supplement: MOVIE S2 [file mSystems.00252-20-sm002.gif]

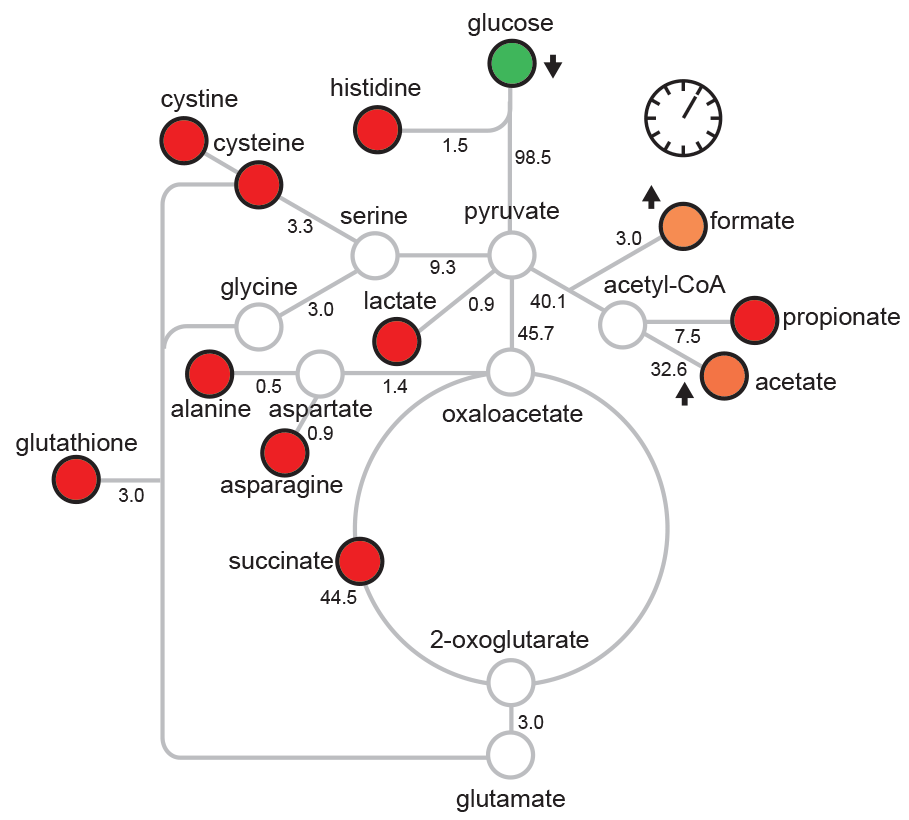

Supplement: MOVIE S3 [file mSystems.00252-20-sm003.gif]
