# Supplementary material for: Metabolic Feedback Inhibition Influences Metabolite Secretion by the Human Gut Symbiont Bacteroides thetaiotaomicron
Source: mSystems. 2020 Sep 1;5(5):e00252-20. doi: 10.1128/mSystems.00252-20 (PMC7470985; doi:10.1128/mSystems.00252-20)
Supplement: TABLE S3 [file mSystems.00252-20-st003.docx]

**Metabolic feedback inhibition influences metabolite secretion by the human gut symbiont *Bacteroides thetaiotaomicron***

Jennie L. Catlett,^1^ Jonathan Catazaro,^2^ Mikaela Cashman,^3^ Sean Carr,^1^ Robert Powers,^2,4^ Myra B. Cohen,^3^ and Nicole R. Buan^1,4,*^

^1^Department of Biochemistry, University of Nebraska-Lincoln, Lincoln, NE, 68588-0664, USA

^2^Department of Chemistry, University of Nebraska-Lincoln, Lincoln, NE, 68588-0304, USA

^3^Department of Computer Science, Iowa State University, Ames, IA 50011-1090, USA

^4^Nebraska Center for Integrated Biomolecular Communication, Lincoln, NE, 68588-0304, USA

* Address correspondence to Nicole R. Buan, [nbuan@unl.edu](mailto:nbuan@unl.edu).

| **Supplementary Table S3. Student’s T-test p values for metabolomics data in Figure 4b.** | | | |
| --- | --- | --- | --- |
| **Metabolite** | **Suppression Coefficient** | | |
|  | **Slope** | **r^2^** | |
| acetate | -0.005 | 0.937 | |
| formate | -0.058 | 0.999 | |
| succinate | -0.015 | 0.949 | |
| propionate | -0.070 | 0.999 | |
| lactate | -0.580 | 1.000 | |
| histidine | -0.688 | 1.000 | |
| cystine | -0.337 | 1.000 | |
| cysteine | -0.289 | 1.000 | |
| asparagine | -0.781 | 1.000 | |
| glutathione | -0.575 | 1.000 | |
| alanine | -1.007 | 1.000 | |
| Data were obtained from 5 biological and 5 technical replicates, n=25.  *: not statistically significant, p>0.01. | | |  |
