# Supplementary material for: Metabolic Feedback Inhibition Influences Metabolite Secretion by the Human Gut Symbiont Bacteroides thetaiotaomicron
Source: mSystems. 2020 Sep 1;5(5):e00252-20. doi: 10.1128/mSystems.00252-20 (PMC7470985; doi:10.1128/mSystems.00252-20)
Supplement: TABLE S4 [file mSystems.00252-20-st004.docx]

**Metabolic feedback inhibition influences metabolite secretion by the human gut symbiont *Bacteroides thetaiotaomicron***

Jennie L. Catlett,^1^ Jonathan Catazaro,^2^ Mikaela Cashman,^3^ Sean Carr,^1^ Robert Powers,^2,4^ Myra B. Cohen,^3^ and Nicole R. Buan^1,4,*^

^1^Department of Biochemistry, University of Nebraska-Lincoln, Lincoln, NE, 68588-0664, USA

^2^Department of Chemistry, University of Nebraska-Lincoln, Lincoln, NE, 68588-0304, USA

^3^Department of Computer Science, Iowa State University, Ames, IA 50011-1090, USA

^4^Nebraska Center for Integrated Biomolecular Communication, Lincoln, NE, 68588-0304, USA

* Address correspondence to Nicole R. Buan, [nbuan@unl.edu](mailto:nbuan@unl.edu).

| **Supplementary Table S4. Student’s T-test p values for metabolomics data in Figure 5a and b.** | | | | | |
| --- | --- | --- | --- | --- | --- |
| **Metabolite** | **Acetate Supplementation (mM)** | | **10mM Acetate + 10mM Formate** | | |
|  | **0** | **10** | **vs 0mM Acetate** | **vs 10mM Acetate** | |
| acetate | 1 | 2.0E-33 | 1.7E-05 | 4.9E-01* | |
| formate | 1 | 1.3E-06 | 2.5E-26 | 4.0E-27 | |
| succinate | 1 | 6.3E-06 | 7.4E-13 | 4.7E-18 | |
| propionate | 1 | 1.6E-23 | 2.1E-27 | 3.7E-04 | |
| lactate | 1 | 1.6E-14 | 4.4E-13 | 3.3E-01* | |
| histidine | 1 | 5.3E-01* | 7.8E-08 | 1.1E-06 | |
| cystine | 1 | 1.4E-01* | 1.1E-07 | 7.9E-06 | |
| cysteine | 1 | 7.9E-06 | 3.8E-08 | 5.3E-02* | |
| asparagine | 1 | 1.5E-10 | 1.8E-09 | 1.8E-01* | |
| glutathione | 1 | 3.8E-01* | 9.5E-06 | 2.2E-06 | |
| alanine | 1 | 1.7E-02* | 7.5E-07 | 5.4E-11 | |
| Data were obtained from 5 biological and 5 technical replicates, n=25.  *: not statistically significant, p>0.01. | | | | |  |
